# Reevaluating fracture prevalence trends in the U.S.: the importance of weighted analysis in NHANES data – Letter to the Editor regarding ‘Trends in prevalence of fractures among adults in the United States, 1999-2020: a population-based study’ by Bin Xu *et al*.

**DOI:** 10.1097/JS9.0000000000000964

**Published:** 2024-05-13

**Authors:** Xianzuo Zhang, Yuan Chen, Wanbo Zhu, Lei Xu

**Affiliations:** a Department of Orthopedics, The First Affiliated Hospital of USTC, Division of Life Sciences and Medicine, University of Science and Technology of China, Hefei; b Department of Orthopedics, The Second Affiliated Hospital of Anhui University of Chinese Medicine, Hefei, Anhui Province; c Department of Orthopedics, Shanghai Jiao Tong University Affiliated Sixth People’s Hospital, Shanghai Jiao Tong University, Shanghai, People’s Republic of China

*Dear Editor*,

I am writing to you regarding the article titled ‘Trends in prevalence of fractures among adults in the United States, 1999-2020: a population-based study’ by Bin Xu *et al*.^1^, published on 3rd November in the *International Journal of Surgery*. As a clinical orthopedist with a focus on epidemiological studies, I have read the article with great interest, particularly the methodology pertaining to the prevalence estimates of hip, wrist, and vertebral fractures among U.S. adults.

The authors presented unadjusted prevalence rates as endorsed by Lesko *et al*.^2^. Their findings indicate a significant increase in wrist and vertebral fractures over two decades, while hip fractures remained stable. Yet, the NHANES (National Health and Nutrition Examination Survey) guidelines advise weighted analyses due to its complex sampling design, which is essential for accurate U.S. population representation – something the unadjusted figures might not fully capture due to the oversampling of certain subgroups^3^. The complex, multistage probability sampling design necessitates the use of weighted analysis to ensure that the estimates are representative of the U.S. population. Unadjusted estimates may not accurately reflect the prevalence due to the oversampling of certain subpopulations within NHANES.

To address this, we have conducted a weighted analysis of the NHANES data from 1999 to 2020. In our weighted analysis of the same NHANES data, there is a discernible rise in overall fracture prevalence, from 8 to 14%. Notably, hip fractures increased from just over 2% to nearly 3%, and wrist fractures surged from ~7 to 11%. Spine fractures also showed a moderate increase. These results, depicted in Figure 1, offer a different perspective on the fracture trends among U.S. adults.

Further examination reveals that adults over 65 are twice as likely to suffer hip fractures as those aged 50–64. There are also marked differences across races and ethnicities, with non-Hispanic Whites exhibiting a higher prevalence of wrist and spine fractures compared to Mexican American and non-Hispanic Black populations (Table 1). These insights align with those of Bin Xu *et al*. and underscore the necessity for fracture prevention strategies tailored to different demographic groups, paving the way for personalized healthcare interventions.

**Table 1:** Prevalence of hip, wrist, and vertebral fractures among U.S. adults 1999–2020.

		Prevalence of fractures		
	*n*	Hip	Spine	Wrist
Overall	180 080 475	1.74	2.43	9.12
Age group, years				
<50	87 283 808	0.67	1.59	8.38
50–64	53 383 789	1.37	2.91	8.98
≥65	39 412 878	3.45	3.06	10.19
Sex				
Men	85 916 021	1.66	2.87	9.99
Women	94 164 454	1.82	2.03	8.31
Race and ethnicity				
Mexican American	13 285 061	1.27	1.74	6.00
Non-Hispanic Black	19 783 567	1.37	1.07	5.88
Non-Hispanic White	126 572 325	2.21	3.33	12.43
Other Hispanic	9 249 192	1.34	2.01	7.27
Other Race – Including Multi-Racial	11 190 330	1.27	2.61	6.37

While Lesko *et al*.’s approach to estimating prevalence at a specific point in time is validated, it may introduce a statistical bias when assessing trends over multiple years. NHANES data’s weighting corrects for sample representation and demographic changes, ensuring that results are nationally representative and consistent over time. These methods are not only standard practice in previous studies^4,5^ but are also extensively documented on the NHANES and CDC (Centers for Disease Control and Prevention) websites (https://wwwn.cdc.gov/nchs/nhanes/tutorials/weighting.aspx).

To conclude, while the study by Bin Xu *et al*. offers valuable insights into fracture prevalence, incorporating data weighting is critical for an accurate portrayal of trends over time. It guarantees the precision, representativeness, and comparability of the findings, thereby enhancing the utility of the research for health trend analysis and policy-making. We hope this will be of interest to clinical orthopedic surgeons, policymakers, and readers of the *International Journal of Surgery*.

## Ethical approval

Not applicable.

## Consent

Not applicable.

## Sources of funding

Not applicable.

## Author contribution

Z.X.: conceptualization, methodology, writing – original draft preparation, and visualization; C.Y.: data curation, writing – review and editing, and visualization; Z.W.: formal analysis and investigation; X.L.: supervision and writing – review and editing.

## Conflicts of interest disclosure

There are no conflicts of interest.

## Research registration unique identifying number (UIN)

Not applicable.

## Guarantor

Zhang Xianzuo and Xu Lei.

## Data availability statement

The datasets generated and analyzed during the current study are available from the corresponding author on reasonable request. The analysis was conducted in accordance with the guidelines provided by the National Health and Nutrition Examination Survey (NHANES), and further information on the dataset can be found at [https://wwwn.cdc.gov/Nchs/Nhanes/].

## Guarantor

Zhang Xianzuo and Xu Lei.

## Acknowledgement

The authors thank to shorten the formal title of this letter. We hope the subtitle “Letter to the Editor regarding ‘Trends in prevalence of fractures among adults in the United States, 1999-2020: a population-based study’ by Bin Xu et al.” not show in the formal name indexed in the databases. When shown in the typeset page, it should also appear in smaller size and in a new line.

## Provenance and peer review

Not invited.
